# Calcium and Boron Foliar Fertilizer to Relieve Cracking of ‘Liuyuezao’ Pummelos

**DOI:** 10.3390/foods14040595

**Published:** 2025-02-11

**Authors:** Kaiyang Du, Han Lin, Qin Luo, Tao Li, Hongyu Wu, Bin Wang, Zhixiong Guo, Tengfei Pan, Wenqin She

**Affiliations:** 1 Institute of Storage, Transportation and Preservation of Horticultural Products, College of Horticulture, Fujian Agriculture and Forestry University, Fuzhou 350002, China; 2 Fujian Key Laboratory of Agro-Products Quality and Safety, Institute of Agricultural Quality Standards and Testing Technology Research, Fuzhou 350003, China

**Keywords:** mineral element, fruit quality, cell wall, cell wall enzyme activity

## Abstract

‘Liuyuezao’ pummelo is highly prone to cracking, which seriously affects its quality. The aim of this study was to illustrate the effect of foliar sprays of calcium (Ca) and boron (B) and their combined treatments on the fruit cracking and quality of ‘Liuyuezao’ pummelos during the fruit expansion period (40–55 days after flowering). Analysis of 12 mineral elements of the pericarp by ICP-MS revealed that the three treatments significantly increased the content of calcium and boron in the corresponding pericarp. These treatments effectively reduced the enzyme activities of pectin methylesterase (PME), polygalacturonase (PG), pectin lyase (PL), β-galactosidase (β-Gal), and cellulase (Cx) in the peel and down-regulated the expression of corresponding cell wall-degrading enzyme genes. Calcium, boron, and their combination treatments reduced water-soluble pectin (WSP) in the peel. Simultaneously, they inhibited the degradation of CDTA-soluble pectin (CSP) and Na₂CO₃-soluble pectin (NSP), thereby stabilizing the cell wall structure. Additionally, these treatments enhanced fruit skin break force (Bf) and elasticity (Ela), ultimately decreasing the fruit cracking rate. Diversification analysis showed that Ca and B elements significantly increased the sugar and vitamin C (Vc) content of ‘Liuyuezao’ pummelo fruits and reduced their organic acid content, thus improving fruit quality. The study provides new ideas on the use of fertilizer interactions to control fruit cracking and improve the quality of the pummelo fruit.

## 1. Introduction

Pummelo (Citrus maxima) belongs to the Citrus genus and is one of the most popular horticultural crops. Many new pummelo varieties have been cultivated through hybridization or bud mutation [1]. The ‘Liuyuezao’ pummelo is an early-maturing strain selected from the ‘Guanximiyou’ bud variant. The ‘Liuyuezao’ pummelo is recognized for ripening 30–60 days earlier than “Guanxi” pummelos and for its softer and more delicate fruit with no granulation [2]. However, its severe cracking during fruit expansion has been a major factor limiting its spread.

Calcium (Ca) and boron (B) are pivotal nutrients in fruit trees, playing crucial roles in enhancing fruit cell wall strength, reducing fruit cracking, and improving fruit quality. Ca is the most abundant mineral in the pericarp cell wall and increases cell wall strength [3]. Foliar spraying of calcium fertilizer during fruit development can effectively inhibit ‘Okitsu No. 58’ citrus fruit cracking [4]. By treating crack-prone grapes with exogenous Ca, cell wall thickness can be effectively enhanced, thus reducing the rate and degree of fruit cracking [5]. Elemental B cross-links with the adjacent rhamnogalacturonan II (RG-II) side-chain A apiosyl residue and enhances the stability of the calcium bridge [6]. The Ca and B contents in the pericarp of indehiscent tomatoes were significantly higher than those of easily cracked tomatoes [7]. Foliar spraying of calcium and boron fertilizers can effectively increase the Ca and B content of fruits and reduce the rate of fruit cracking [8]. Ca and B also play an important role in enhancing fruit quality. Calcium spraying improves the sugar–acid ratio of ‘Okitsu No.58’ citrus fruits [4]. Boron increases fruit weight, soluble solids, vitamin C (Vc), and sugar content [9]. Foliar sprays of calcium and boron fertilizers significantly increase soluble sugar content in apple fruits [10]. Calcium–boron mix increases Vc content in cherry tomatoes [11].

As the direct site of fruit dehiscence, abnormal cell wall metabolism is a key factor leading to dehiscence, and cell wall composition and associated enzyme activities are closely related to fruit dehiscence resistance. The main components of the cell wall consist of pectin-like polysaccharides, cellulosic polysaccharides, hemicellulosic polysacc-harides, etc. [12,13]. Crack-resistant varieties were found to contain higher levels of CSP, NSP, and hemicellulose (HC), and lower levels of WSP compared to crack-prone varieties of grape [10]. The CSP and NSP contents of Ponkan fruit were significantly increased, and WSP was suppressed in the rind after Ca treatment [14]. Hydrolysis of cell wall components is regulated by a range of enzymes. Pectin methylesterase (PME), polygalacturonase (PG), and cellulase (Cx) activities in the pericarp of cleavage-prone varieties were found to be significantly higher than those of cleavage-resistant varieties from the study of litchi [15]. The application of calcium fertilizer to ‘Xiangfei’ grapes during fruit turnout resulted in a reduction in the activities of PME, pectin lyase (PL), PG, β-galactosidase (β-Gal), and Cx, as well as a decrease in the expression levels of related genes [16]. Grapefruit PG, PME, and Cx activities increased to varying degrees during post-harvest storage, and the corresponding gene expression levels also increased to varying degrees [17].

This study employs foliar sprays of Ca and B, as well as their combined treatments, on the fruit fissure-prone ‘Liuyuezao’ variety pummelo to ascertain the impact of different fertilization regimes on cell wall components and cell wall-degrading enzyme activities and their related gene expression, as well as the mechanical properties of the peel and fruit quality of pummelo fruit peels. This study aimed to elucidate the relationship between calcium and boron elements and the metabolism of cell wall substances in pummelo fruit and also facilitate the elucidation of the factors influencing fruit cracking, which can provide a theoretical basis for improving fruit quality.

## 2. Materials and Methods

### 2.1. Test Materials and Treatments

‘Liuyuezao’ pummelos were used as the test material, which were collected from a pummelo orchard in Wuzhai Township, Pinghe County, Zhangzhou City, Fujian Province, China. Thirty-six ‘Liuyuezao’ pummelo trees of uniform size and vigorous growth were randomly selected in the orchard, and three neighboring trees were set as one treatment and repeated three times. The fertilizers calcium chelate (Brandt, Tampa, FL, USA) and borax (Borax, Boron, CA, USA) were used to provide Ca and B, respectively. The fruits and leaves of each plant were treated with a solution of 0.1% fertilizer, both at 40 days after flowering (40DAF) and 55 days after flowering (55DAF), which coincided with the period of fruit expansion. The study employed three treatment groups: Ca foliar fertilizers, B foliar fertilizers, and a combination of both (Ca + B). Water was used as the control check (CK). The samples were harvested at 45 days after treatment (100 DAF), 55 days after treatment (110 DAF), 65 days after treatment (120 DAF), 75 days after treatment (130 DAF), and 85 days after treatment (140 DAF). Ten fruits of uniform size were randomly selected from each tree. Then, the fruits were washed and the flesh and peel were subsequently separated. Samples were stored at −80 °C until the subsequent analysis was conducted. Fruit cracking rate (FCR) was also determined, and FCR was represented as number of cracked fruits per plant/total number of fruits per plant ×100%.

### 2.2. Mineral Elements

We accurately weighed and transferred 0.3 g of peel into an ablution vessel and added 5 mL of nitric acid solution. Following an interval of one hour, the samples were subjected to digestion using a TOPEX microwave digestion system (Yi Yao, Shanghai, China). After the solution cooled to 20 °C, it was transferred into a volumetric flask, and the volumes were set to 25 mL with deionized water. The mineral content of the peel was subsequently determined using an inductively coupled plasma mass spectrometer (ICP-MS) model 2030 (Shimadzu, Tokyo, Japan) [18]. Results are expressed as mg/kg.

### 2.3. Fruit Phenotype and Sugar-Acid Content

The quality of the fruits at maturity (140 DAF) was evaluated. The individual fruits were weighed using an electronic scale. The longitudinal and transverse diameters of the fruit were measured at the largest point of the girdle using vernier calipers, and the fruit shape index was the value of the longitudinal diameter over the transverse diameter [19]. Sucrose (Su), fructose (Fr), glucose (Glu), citric acid (Cia), malic acid (Maa), Vc, shikimic acid (Sha), quinic acid (Qua), and α-ketoglutaric acid (Kea) were assessed using UPLC-ESI-MS/MS. The sum of the contents of Su, Fr, and Glu represented the total sugars (TS), while the sum of the contents of the six acid fractions represented the total acids (Ora) [1]. The ratio of sugars to acids was the sugar–acid ratio (Gra). Results are expressed as mg/g.

### 2.4. Mechanical Properties of Peel

Three fruits were randomly selected from each tree, and nine points were randomly selected from each fruit for puncture experiment. The experiment was conducted using a compact benchtop universal/tensile tester (Shimadzu, Japan). The maximum puncture force leading to rupture of the pericarp was the skin Bf, results expressed as N, and the deformation distance before rupture of the pericarp was the pericarp Ela [20], and the results were expressed as mm.

### 2.5. Cell Wall Polysaccharide and Cell Wall Enzyme Activity

The extraction of the crude cell wall of the pericarp and the isolation of WSP, CSP, NSP, HC, and cellulose (CEL) were conducted in accordance with the methodology of Vicente et al. [21]. The sum of WSP, CSP, and NSP was total pectin (TP). Determination of pectin, HC, and CEL according to previously published methods [22]. Both pectin and cellulose contents are expressed in mg/g. The plant pectin methylesterase (PME) activity, plant polygalacturonase (PG) activity, plant pectin lyase (PL) activity, plant β-galactosidase (β-Gal) activity, and plant cellulase (Cx) activity were measured following the manufacturer’s instructions of an assay kit (COIBO, Shanghai, China). Several units of enzyme activity are expressed in U/g.

### 2.6. Real-Time Fluorescence Quantitative PCR Analysis of Cell Wall-Degrading Enzyme Gene Expression

Five genes that exhibited differential expression in relation to cell wall modification were selected through the pre-transcriptomic data of our group. These genes were PME (Cg1g013250), PG (Cg5g041660), PL (Cg5g006150), β-Gal (Cg8g020510), and Cx (Cg4g016620). Primers were designed using the Primer Primer 5.0 software. The citrus actin gene was used as an internal reference gene, and the primer sequences are presented in (Table S1). Total RNA was extracted using the CTAB method with minor modifications [23]. RNA was reverse-transcribed into cDNA using the Hifair^®^ III Reagent Kit 1st strand cDNA Synthesis SuperMix for qPCR Reverse Transcriptase (Yeasen, Shanghai, China). The polymerase chain reaction (PCR) system and procedure were based on the specifications of the 2 × RealStar Green Fast Mixture (GenStar, Beijing, China). qPCR analyses were performed on a qTOWER3 real-time PCR thermocycler (Analytik Jena AG, Jena, Germany).

### 2.7. Statistical Analyses

The study utilized Microsoft Excel 2019 for data organization and IBM SPSS Statistics 27.0 software for data analysis. First, the data set was tested for normality using the Shapiro–Wilk test, to ensure that the data statistically met the normality assumption. Subsequently, the variance alignment of the data was assessed through the Levene test. Subject to the fulfillment of normality and variance alignment, the data were finally analyzed further statistically using a one-way analysis of variance. (Duncan test; different letters in the graphs indicate statistical significance at the 0.05 level). Correlation heatmaps and correlation networks were constructed using the OmicStudio tool (https://www.omicstudio.cn/tool (accessed on 12 September 2024)). Principal component analysis (PCA) and orthogonal partial least squares discriminant analysis (OPLS-DA) were conducted using the OmicShare tool (https://www.omicshare.com/tools/ (accessed on 15 September 2024)) [24].

## 3. Results

### 3.1. Changes in Rate of Cracking of Pummelo ‘Liuyuezao’ Fruits

**Figure 1:**
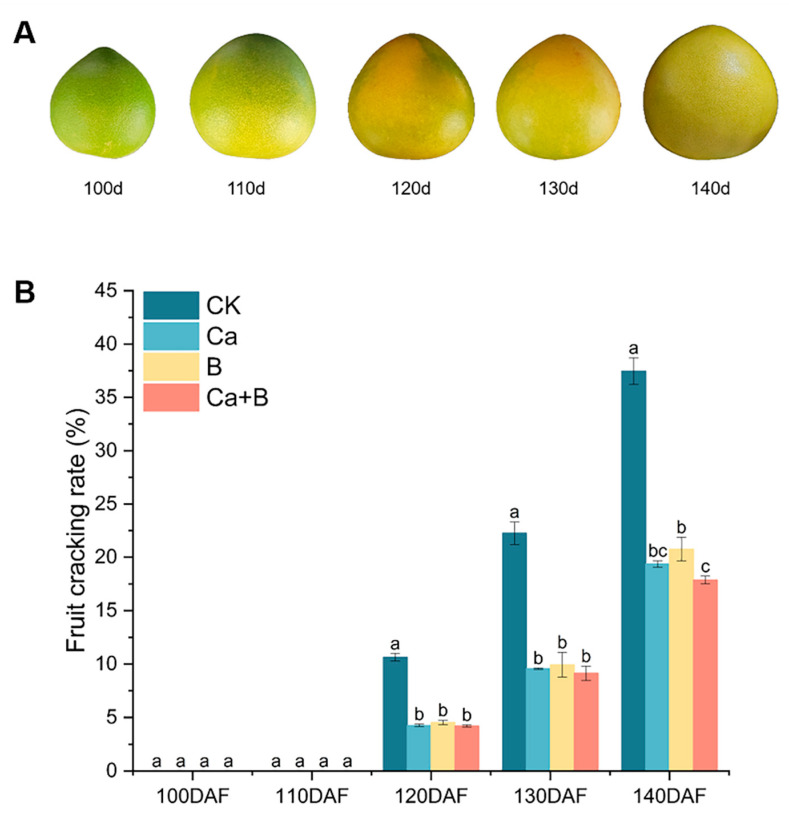
(**A**) Development of ‘Liuyuezao’ pummelo fruits; (**B**) effect of different treatments on cracking rate of ‘Liuyuezao’ pummelo fruits. Parameter values shown in graphs are expressed as mean ± standard error (n = 3). Different letters indicate statistical differences between treatments (Duncan test, *p* < 0.05).

The fruit condition and fruit cracking rate of ‘Liuyuezao’ pummelos at different developmental periods are shown in Figure 1. Fruit cracking started at 120 DAF, followed by a sharp increase in the rate of cracking, which peaked at fruit maturity (140 DAF). The incidence of fruit splitting was markedly reduced in the treatment groups in comparison to the control group at each subsequent stage following the onset of fruit splitting. At 140 DAF, the cracking rate of the Ca + B treatment was 19.6% lower than that of the CK. This suggests that Ca or B foliar fertilizers have the potential to mitigate fruit cracking in ‘Liuyuezao’ pummelo.

### 3.2. Mineral Content of ‘Liuyuezao’ Pummelo Pericarp

The findings revealed a notable elevation in aluminum (AI) content at 110–120 days and 140 days under Ca treatment. Both Ca and Ca + B treatments significantly promoted Ca accumulation in the pericarp. The B and Ca + B treatments were found to play a significant role in promoting B enrichment in the pericarp. A significant decrease in copper (Cu) content was observed in all treatment groups at 130 days. The concentration of iron (Fe) in the pericarp was markedly elevated in the Ca + B treatment in comparison to the control throughout the developmental phase. The kalium (K) content of the pericarp was significantly higher in the Ca + B treatment group than in the CK at 120 DAF. The Ca and Ca + B treatments were observed to significantly promote pericarp magnesium (Mg) accumulation at 120–130 DAF There were no significant differences in the manganese (Mn), sodium (Na), nickel (Ni), zinc (Zn), and selenium (Se) contents in the pericarp of the treatment groups compared to the CK (Table S2).

**Figure 2:**
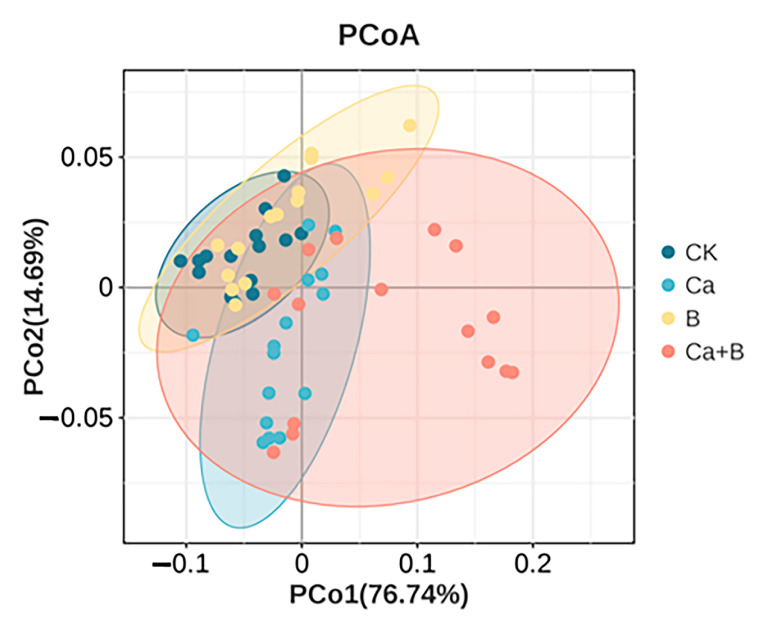
PCA scores of mineral elements of ‘Liuyuezao’ pummelo pericarps.

**Figure 3:**
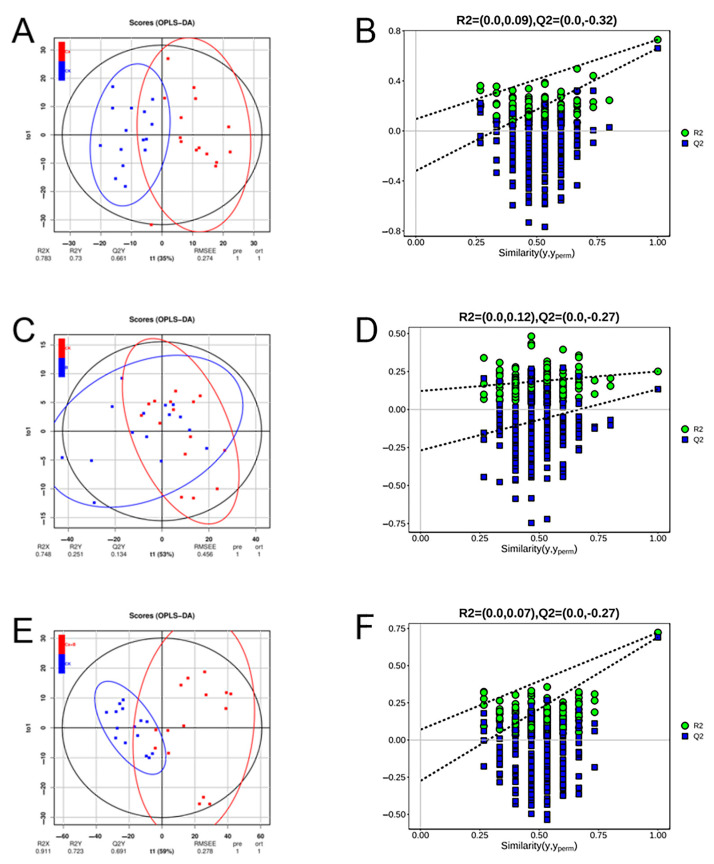
Orthogonal partial least squares discriminant analysis of the mineral content of ‘Liuyuezao’ pummelo peel. (**A**–**F**) Plots of OPLS-DA scores and OPLS-DA statistical model ranking test validation for mineral elements under CK vs. Ca, B, and Ca + B treatments, respectively.

In this study, a principal component analysis (PCA) was conducted on 12 mineral elements, with 60 samples from 4 treatments across 5 periods. The resulting scatter plots of the principal components of the mineral elements were then obtained (Figure 2). The combined variance of PCo1 and PCo2 accounted for 91.43% of the total variance, with significant clustering. OPLS-DA is a supervised multivariate projection method that combines Orthogonal Signal Correction (OSC) and partial least squares discriminant analysis (PLS-DA) to screen for variance variables by removing uncorrelated variances. It is primarily employed for correlation and discriminant analysis. The mineral elements were subjected to OPLS-DA testing in the CK and the three treatment groups, respectively. We chose variables with VIP (Variable Importance in Projection) ≥ 1, a *p* value from a univariate statistical analysis (*t*-test) < 0.05, and a multiplicity of difference equal to 1 for screening purposes. Mineral content in the pericarp was well segregated between groups for CK, Ca and Ca + B treatment groups. The intersection of the Q^2^ regression line with the vertical axis is less than zero, indicating that the model is not overfitted and is therefore valid (Figure 3). This indicates that the results can be used for the analysis of mineral element identification in the peel of the CK group and the Ca and Ca + B treatment groups, respectively.

### 3.3. Fruit Phenotypes and Sugar–Acid Content of ‘Liuyuezao’ Pummelos

Fruit weight, fruit longitudinal and transverse diameter, and fruit shape index did not differ significantly from the CK after treatment. The Su, Fr, and Glu of fruits were significantly higher under Ca + B treatment than in the CK. The TS content of the fruits was significantly higher in all three treatments. Cia content decreased significantly after the B and Ca + B treatments, whereas Vc content increased significantly. Subsequent analysis demonstrated a notable decline in the levels of Maa, Sha, and Ora following the application of all three treatments. In addition, the Gra of the fruits was all shown to be higher after spraying with foliar fertilizer (Table S3).

**Figure 4:**
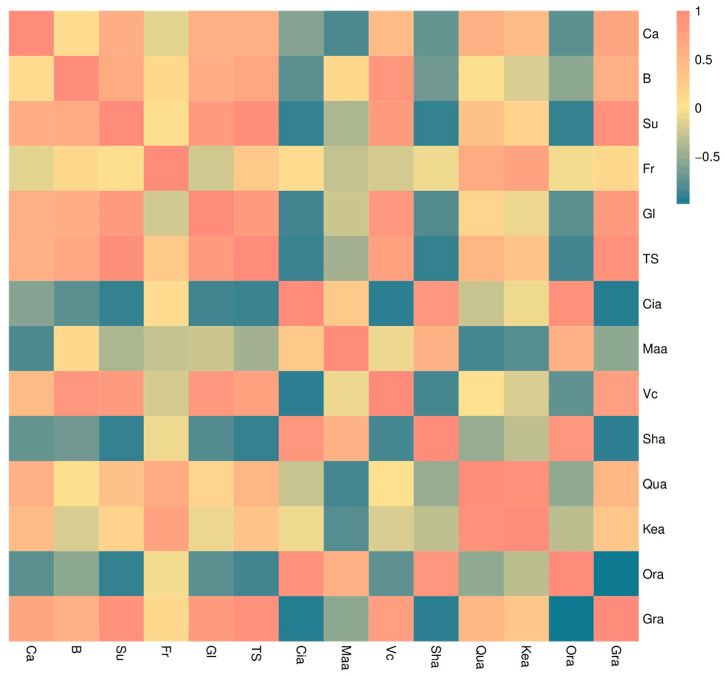
The correlations between Ca and B and the content of sugar and acid fractions of the fruit.

The analysis shows that Ca has a significant positive correlation with Su, Glu, Qua, and Gra, but a significant negative correlation with Maa, Sha, and Ora (Figure 4).

### 3.4. Changes in Skin Break Force and Elasticity of ‘Liuyuezao’ Pummelos

**Figure 5:**
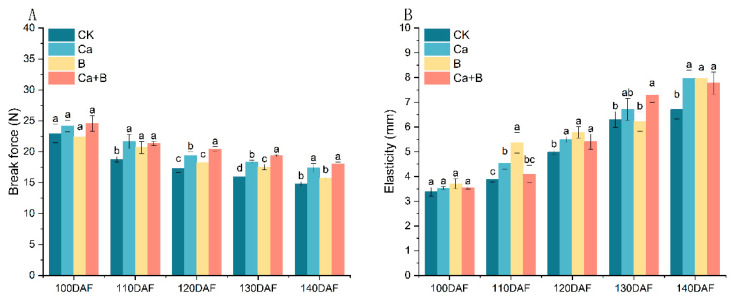
(**A**) Effect of different treatments on skin break force of ‘Liuyuezao’ pummelos; (**B**) Effect of different treatments on skin elasticity of ‘Liuyuezao’ pummelos. Parameter values shown in graphs are expressed as mean ± standard error (n = 3). Different letters indicate statistical differences between treatments (Duncan test, *p* < 0.05).

The Bf of the skin of the ‘liuyuezao’ pummelos decreased gradually throughout the stage. From 110 DAF onwards, the Bf of the skin was markedly greater in the Ca and Ca + B treatments than in the CK treatment. Pericarp Ela rose with time from 100 DAF. At 120 DAF and 140 DAF, the peel Ela of the three treatments was significantly improved compared to the CK (Figure 5).

### 3.5. Changes in Polysaccharide Content of Pericarp Cell Wall of ‘Liuyuezao’ Pummelos

**Figure 6:**
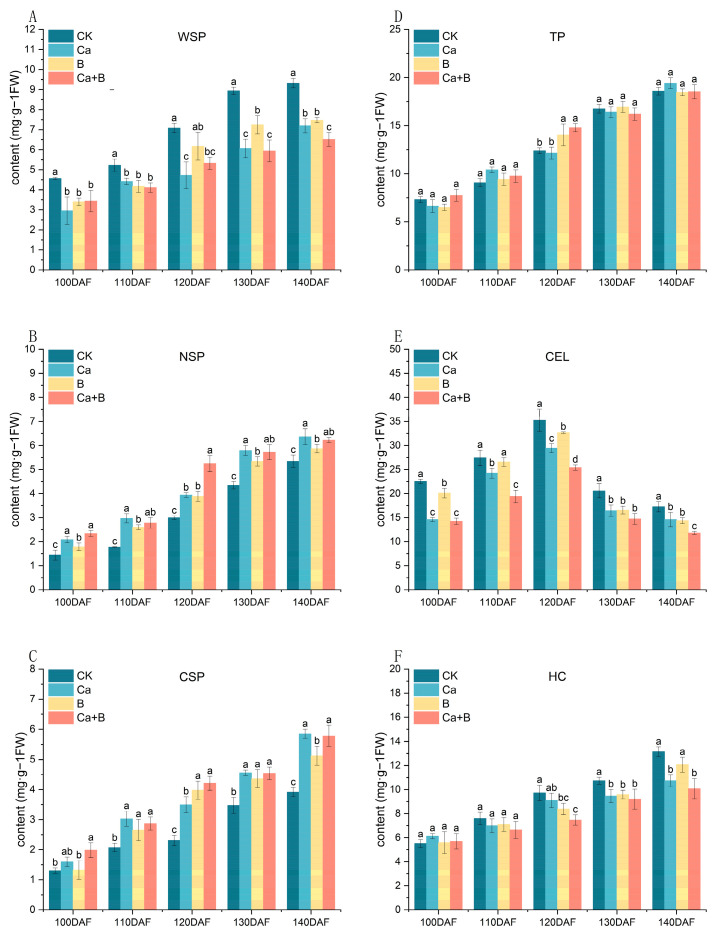
A comparative analysis of the polysaccharide composition of the pericarp cell wall of the ‘Liuyuezao’ pummelo fruit, as influenced by different treatments. (**A**) WSP content, (**B**) NSP content, (**C**) CSP content, (**D**) TP content, (**E**) CEL content, (**F**) HC content. Parameter values shown in the graphs are expressed as mean ± standard error (n = 3). Different letters indicate statistical differences between treatments (Duncan test, *p* < 0.05).

The concentrations of WSP, CSP, NSP, TP, and HC in the pericarp of the ‘Liuyuezao’ pummelos exhibited an increase with fruit development, whereas the concentration of CEL demonstrated an initial increase followed by a subsequent decrease. The WSP content of the CK treatment group was markedly higher than that of the Ca treatment group in each period. Following the onset of fruit cracking (120 DAF), the CSP content was found to be significantly higher in all three treatments in comparison to the CK. At 140 DAF, the Ca treatment exhibited a 49.47% increase in CSP content relative to the CK. The Ca and Ca + B treatments were found to significantly promote NSP content at each stage of the experiment. There were no significant differences in TP content between the treatments and CK at the 100–110 days after anthesis (DAF) and 130–140 days after flowering (DAF) time points. The CEL content of Ca and Ca + B-treated pericarps was significantly reduced. The Ca and Ca + B treatments were found to significantly increase HC content at 140 DAF (Figure 6).

### 3.6. Expression of Cell Wall Enzymes and Genes Related to Cell Wall Metabolism in Pericarp of ‘Liuyuezao’ Pummelos

**Figure 7:**
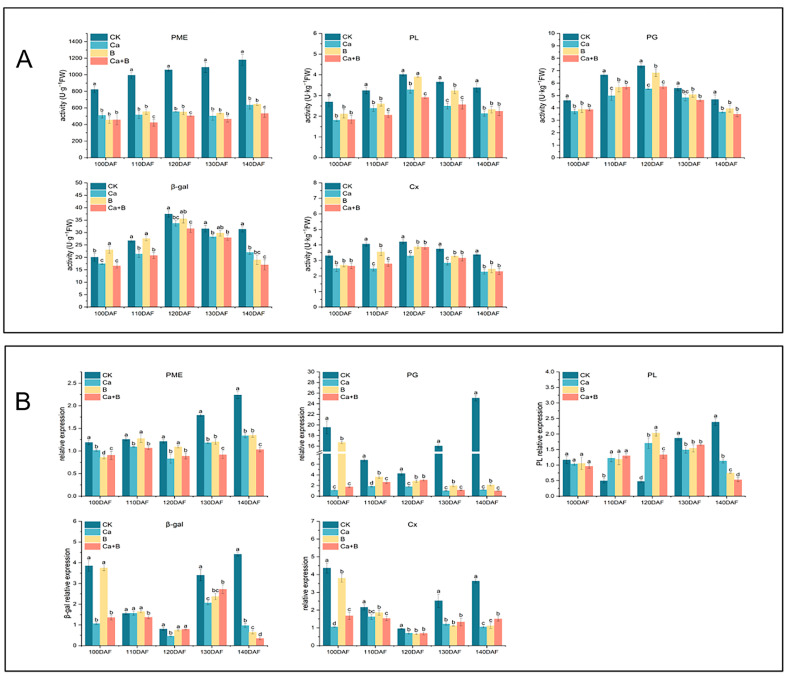
(**A**) Comparison of cell wall enzyme activities in pericarp of ‘Liuyuezao’ pummelos by different treatments; (**B**) effects of different treatments on qRT-PCR of genes related to cell wall metabolizing enzymes in pericarp of ‘Liuyuezao’ pummelos. Parameter values shown in graphs are expressed as mean ± standard error (n = 3). Different letters indicate statistical differences between treatments (Duncan test, *p* < 0.05).

At each stage, PME activity in the pericarp of the CK group was significantly increased, whereas the increase in PME activity was significantly suppressed in the three treatments, with a difference of 647.01 (U·g-1FW) between the Ca + B treatment and the CK at 140 DAF. The activities of PG, PL, β-gal, and Cx exhibited a pattern of initial increase followed by a decline throughout the growth phase. The growth of PG activity during these periods at 100–120 DAF and 140 DAF was markedly suppressed under all three treatments, resulting in a notable reduction in the level of PG activity. PL activity was significantly reduced in the peel of the three treatments during 130–140 DAF. The Ca + B treatment consistently exhibited markedly diminished β-gal activity in comparison to the CK group across all tested periods. Furthermore, β-gal activity was notably reduced in all three treatments at 140 DAF. During fruit development, Cx activity was significantly reduced by both the Ca and Ca + B treatments (Figure 7A).

There was a notable reduction in the expression of PME in response to both the Ca and Ca + B treatments. PG was significantly down-regulated in expression by all three treatments at 100–140 DAF. The expression of PL was significantly up-regulated by the three treatments at 110–120 DAF and significantly down-regulated at 130–140 DAF. β-gal was subject to a successive down-regulation in expression by all three treatments at 130–140 DAF, with a significant 12.95-fold down-regulation being observed at 140 DAF as a result of the Ca + B treatment. Cx was significantly down-regulated in expression by all three treatments throughout the period (Figure 7B).

## 4. Discussion

Foliar sprays of Ca and B fertilizers can effectively improve fruit crack resistance and significantly reduce the rate of fruit cracking [12]. In this study, we found that fruit cracking rate decreased significantly after Ca and B fertilizer application, with the most significant effect being that of Ca + B treatment at 140 DAF (Figure 1B). Further analysis revealed that the Ca or B content of the peel was significantly enriched by spraying the corresponding fertilizer, and when Ca and B were mixed, their elemental content showed superior results compared to a single application (Table S2). This is consistent with the results of previous Ca fertilization trials on rutabagas and grapes, both of which showed that Ca supplementation had a positive effect on enhancing fruit crack resistance [14,16]. In addition, the mixed application of B and Ca nitrate was also shown to be effective in reducing fruit cracking in pomegranates, further confirming the synergistic effect of Ca and B elements in the enhancement of fruit cracking resistance [8].

Previous studies have found that spraying Ca or B can improve the intrinsic quality of fruit [10]. The results demonstrated that the foliar application of Ca or B had no notable impact on the fruit weight, longitudinal and transverse diameter, and fruit shape index of ‘Liuyuezao’ pummelos (Table S3), which was in alignment with the findings of previous studies on kiwifruit and strawberries [25,26]. It is suggested that the effects of these elements on fruit morphology may vary from species to species. The experimental results demonstrated that the Ca + B treatment was the most effective in enhancing fruit quality, with a significant increase in the Su, Fr, Glu, and Vc content of the fruit, and a significant reduction in the organic acid content of Cia, Maa, Sha, and Qua (Table S2). Subsequent analyses indicated a close relationship between the elemental composition of the pericarp and these quality indicators. The Ca and B contents demonstrated significant positive correlations with the Su, Glu, and Gra of the fruits, while B exhibited significant positive correlations with the TS and Vc contents of the fruits. In contrast, Ca demonstrated significant negative correlations with the Maa, Sha, and total acid contents, while B exhibited significant negative correlations with the Cia and Sha contents (Figure 4). Similar effects have been demonstrated in other fruits. The application of Ca and B sprays resulted in an increase in the TS content of the ‘Fuji’ and ‘Orin’ apples [10]. Ca fertilizer sprayed on ‘Okitsu No. 58’ citrus reduced the content of organic acids in the fruit [4]. Furthermore, the addition of B led to an enhancement in the soluble solids, Vc, and TS content of strawberry fruits [27]. In the present experiment, a substantial enhancement in the physical characteristics of the peel was also observed. All three treatments significantly increased peel Bf and Ela (Figure 5), which is consistent with the results observed after Ca treatments on apples and grapes [27,28]. Although Ca and B sprays did not have a significant effect on the morphology of ‘Liuyuezao’ pummelo fruits, they showed a significant potential to improve fruit quality, especially when used in combination.

Cell wall components and cell wall metabolic processes are closely related to fruit splitting [29,30]. The three treatments employed in this experiment were found to significantly reduce WSP and to significantly increase peel CSP and NSP content. The TP content of the pericarp was found to be significantly higher in the Ca + B treatment at the early stage of fruit splitting, although no significant difference was observed between this treatment and the CK at the later stage. In addition, we found that the CEL content of the Ca and Ca + B treatment groups started to decrease significantly at the stage of fruit splitting (Figure 6). Ca and B synergize to build plant cell walls in tomato plants [31]. Ca^2+^ and B can connect with pectin, thus making the cell wall stronger. This mechanism was demonstrated in the crack-resistant tomato variety ‘L1698’, which had significantly higher contents of protopectin (CSP and NSP) as well as HC than the crack-prone variety ‘L2683’, while its WSP content was relatively low [7]. Similarly, a highly significant negative correlation between protopectin content and fruit splitting rate was found in the study of Jincheng oranges [32].

The results of this experiment demonstrate that the activities of PME, PG, PL, β-Gal, and Cx in the pericarp were significantly inhibited to varying degrees under the three treatments. This has a beneficial impact on the slowing down of the degradation of cell wall substances in the pericarp, thus maintaining the structural integrity of the cell wall. The experimental results also showed that the inhibitory effect of different fertilization treatments on the activity of degradative enzymes exhibited a significant time dependence (Figure 6) The activity of the PME was subject to significant regulation under all three treatments throughout the period in question. At 100–120 DAF, PG activity was significantly suppressed under all three treatments. Pericarp PL activity was significantly diminished under all three treatments relative to the CK during the 130–140 DAF period. It is noteworthy that the Ca + B treatment was particularly significant in modulating β-gal activity throughout the experimental cycle. Moreover, a significant reduction in Cx activity was observed throughout the period under the Ca treatment. Previous studies have confirmed that higher PME, PG, β-Gal, and CX activities may be the main cause of fruit cracking in navel oranges [33], whereas significant reductions in PG, PME, PL, and β-gal activities were also observed in ‘Fuji Kiku-8’ apples, ‘Okitsu No. 58’ citrus, and Ponkan fruits in experiments where foliar sprays of calcium fertilizers were carried out during the fruit growth period [4,5,34]. The degradation of pectin is a complex process involving the synergistic action of several enzymes. Among other things, PME is capable of removing methyl ester groups from pectin side-chains, thereby providing a usable substrate for the degradation of PG and PL [13,35,36]. β-Gal plays an important role in the degradation of side-chain glycosidic branched chains of pectin and hemicellulosic polysaccharides. By inhibiting the activity of β-Gal, these side-chain moieties can be effectively protected against the exposure of pectin-like degrading enzymes to these substrates [37,38,39]. Cx has been demonstrated to possess the capacity to degrade polysaccharides with a glucan structure, including CEL and HC [40,41].

The gene expression trends of PME, PG, and Cx in the pericarp were found to be generally consistent with the observed changes in enzyme activities, indicating that the changes in gene expression levels determined the enzyme activities to some extent. However, β-gal and PL gene expression converged with the expression of enzyme activity at the late stage and differed at the early stage, which may reflect spatial and temporal differences between enzyme activity and gene expression, or the gene family may have different expression characteristics at different stages of pericarp dehiscence [17]. Further analysis showed that Ca and B treatments vary in the stage of regulation and inhibition of the expression of hydrolytic enzyme genes in the cell wall of the pericarp. The expression of PME genes was markedly repressed by the Ca and Ca + B treatments throughout the developmental period. Similarly, the expression of PG and Cx genes was significantly repressed by all three treatments throughout the developmental period. Moreover, the expression of PL and β-gal genes was significantly repressed by all three treatments at 130–140 DAF.

In conclusion, the application of Ca and B treatments by foliar spraying during the fruit development phase can reduce the incidence of cracking by regulating the expression level of genes encoding cell wall-degrading enzymes and their associated enzyme activities. The combination treatment of Ca and B showed a more pronounced effect than the single treatments of Ca and B.

## 5. Conclusions

Ca, B, and a mixture of both treatments were able to significantly increase the Ca and B content of fruit pericarps. Ca + B treatment was the most effective in improving fruit quality as it significantly increased Su, Fr, Glu, and Vc contents while reducing acid content. The three treatments significantly inhibited the activity and related gene expression of PME, PG, PL, β-Gal, and Cx in the pericarp, which resulted in lower WSP content and significantly higher CSP and NSP content in the pericarp, enhanced pericarp Bf and pericarp Ela, and reduced the rate of fruit splitting. Based on the results of the above experiments, we conclude that the foliar spraying of calcium or boron and the combination of these two elements can significantly reduce the rate of fruit splitting in ’Liuyuezao’ pummelos, which is important for improving the yield and quality of pummelo fruits. However, despite the fact that we have achieved some important research results, the exploration of potential genetic variation for the breakage-prone trait in pummelos is still insufficiently advanced. Therefore, this is an important direction that needs to be further strengthened in the future to better understand the genetic mechanism of pummelo fruit cracking and to provide strong theoretical support for breeding pummelo varieties with a greater resistance to cracking.

## Data Availability

The original contributions presented in the study are included in the article, further inquiries can be directed to the corresponding author.

## Supplementary Materials

The following supporting information can be downloaded at: https://www.mdpi.com/article/10.3390/foods14040595/s1, Table S1: Primer sequences for ‘Liuyuezao’ pummelo; Table S2: Content of mineral elements in the rind of ‘liuyuezao’ pummelo; Table S3: Fruit phenology and sugar and acid profiles of ‘liuyuezao’ pummelo after spraying with Ca or B and its mixture.
