# Supplementary material for: Calcium and Boron Foliar Fertilizer to Relieve Cracking of ‘Liuyuezao’ Pummelos
Source: Foods. 2025 Feb 11;14(4):595. doi: 10.3390/foods14040595 (PMC11854133; doi:10.3390/foods14040595)
Supplement: Supplementary file 1 [file foods-14-00595-s001.zip › foods-3453736-supplementary.pdf]

Table S1．Primer sequences for 'Liuyuezao' pummelo

Gene name	Primes sequence (5′-3′)	
	**F**	**R**
PME	ACACACTCTACGTCCACTCG	GCTTTCGTGCATGGATGTCA
PG	AAACCAGGGCCAGTACTCTC	CTGAAACTCCCACAAGTGCC
PL	GGGACTTGATGGTGAACGGT	ACAAGTGCCGATGGTCTAGC
β-Gal	CGGATGGGACGAGTATGGAA	ACGTCTTGAATCCCACCTGA
Cx	TGGCCTTCACGACAACAATG	TTGAGGAGATAGTCGGTCGC
Actin	CCATGTACGTTGCCATCCAG	ACCATCACCAGAATCCAGCA

Table S2. Content of mineral elements in the rind of 'liuyuezao' pummelo.

	CK	Ca	B	Ca+B
AI（100DAF）	16.3±1.228a	15.4±0.458a	14.766±1.357a	14.466±1.154a
AI（110DAF）	10.646±1.772c	17.233±0.305a	13.6±0.721b	8.946±1.175c
AI（120DAF）	24.966±2.196b	37.6±3.218a	24.466±1.05b	16.533±0.321c
AI（130DAF）	15.3±2.487a	10.823±1.176b	14.4±0.721a	15.566±2.281a
AI（140DAF）	15.966±1.011c	27.8±1.153a	16.7±0.953c	21.366±1.686b

B（100DAF）	3.493±0.118b	3.276±0.207b	5.423±0.116a	5.36±0.16a
B（110DAF）	3.386±0.153b	3.348±0.221b	4.833±0.09a	4.8±0.117a
B（120DAF）	3.203±0.085b	2.743±0.096c	4.576±0.075a	4.73±0.229a
B（130DAF）	2.456±0.222c	2.283±0.181c	3.513±0.209b	3.9±0.13a
B（140DAF）	2.47±0.235b	1.986±0.117c	3.673±0.19a	3.89±0.121a
Ca（100DAF）	530±10c	799.333±19.139b	552.333±21.385c	856.666±14.64a
Ca（110DAF）	532.333±10.969c	741.333±28.29b	516.666±23.028c	852.333±34.122a
Ca（120DAF）	441.666±19.218c	642.666±18.009b	459.333±41.932c	743.333±22.546a
Ca（130DAF）	434.333±21.939b	630.666±43.924a	438±20.223b	598.333±31.342a
Ca（140DAF）	375.333±28.936b	584±45.825a	420±18.248b	621±36.29a
Cu（100DAF）	18.233±2.227a	21.033±4.742a	18.333±1.792a	18.9±2.066a
Cu（110DAF）	16.333±0.115a	19.1±2.594a	19.333±3.524a	16.2±0.2a
Cu（120DAF）	19.7±0.173a	19.466±0.321a	17.766±1.209a	18.333±1.75a
Cu（130DAF）	21.7±2.286a	16.366±0.152b	17.633±2.309b	16.766±0.057b
Cu（140DAF）	18.466±3.146a	21.7±4.386a	16.133±0.057a	17.6±1.819a
Fe（100DAF）	44.8±5.702a	61.133±7.02a	48.466±12.577a	49.033±6.53a
Fe（110DAF）	33.033±3.837b	56.066±6.709a	51.8±5.548a	27.366±1.379b
Fe（120DAF）	50.8±6.614b	66.033±8.5a	43.8±7.989b	50±6.533b

Fe（130DAF）	57.366±9.279a	52.733±6.143ab	40.933±9.385b	43.066±4.66ab
Fe（140DAF）	41.566±6.388b	64.933±4.31a	37.7±9.676b	46.033±1.331b
K（100DAF）	2356.666±140.119c	2313.333±90.737c	3050±173.493b	3793.333±134.288a
K（110DAF）	2230±60.827b	2230±30b	2203.333±45.092b	2330±52.915a
K（120DAF）	2220±147.309b	2353.333±97.125b	2416.666±214.553b	3443.333±477.214a
K（130DAF）	2136.666±196.553a	2500±216.333a	2436.666±215.947a	2463.333±185.831a
K（140DAF）	2363.333±174.737b	2370±407.308b	2450±170.88b	3193.333±555.817a
Mg（100DAF）	428±29.103ab	349±25.239b	436.667±48.789a	465±10.392a
Mg（110DAF）	338.667±1.528a	342.667±4.163a	342±14.526a	331.667±12.583a
Mg（120DAF）	334.333±27.538b	413.333±18.824a	368±31.749ab	409.667±16.166a
Mg（130DAF）	337.333±38.695c	430.667±26.764ab	378.667±43.616bc	451.333±25.325a
Mg（140DAF）	361.667±23.159a	365.333±4.509a	383.333±27.301a	382.333±14.189a
Mn（100DAF）	12.067±0.153a	12.333±0.709a	11.867±0.351a	12.067±0.513a
Mn（110DAF）	10.833±0.153ab	11.3±0.529ab	11.367±0.451a	10.633±0.058b
Mn（120DAF）	11.5±0a	12.1±0.265a	11.4±0.173a	11.9±0.173a
Mn（130DAF）	11.8±0.557a	11.5±0.2a	11.567±0.351a	11.9±0.2a
Mn（140DAF）	11.067±0.462a	11.9±1.114a	11.2±0.2a	11.133±0.404a
Na（100DAF）	355.333±8.737a	458.333±27.429a	425±61.441a	374±10.44a

Na（110DAF）	478.667±7.024a	475.333±4.509a	435.667±14.189a	435±25.357a
Na（120DAF）	335.333±9.019a	386.333±40.464a	347.667±17.214a	354.333±10.408a
Na（130DAF）	443±17.578a	441.667±17.01a	436.333±12.662a	435.333±3.215a
Na（140DAF）	395±15.716a	389±58.924a	433±66.731a	397±11.533a
Ni（100DAF）	0.753±0.119a	0.86±0.295a	0.881±0.373a	1.073±0.525a
Ni（110DAF）	0.526±0.046a	0.644±0.171a	0.637±0.004a	0.641±0.188a
Ni（120DAF）	0.957±0.376a	1.078±0.46a	0.82±0.268a	0.812±0.038a
Ni（130DAF）	0.666±0.092a	1.254±1.172a	0.649±0.209a	0.775±0.198a
Ni（140DAF）	0.591±0.146a	0.813±0.304a	0.7±0.12a	0.859±0.392a
Zn（100DAF）	4.223±0.652a	4.353±0.379a	2.46±0.419a	5.717±0.238a
Zn（110DAF）	3.86±0.858a	4.263±0.355a	4.153±0.802a	2.43±0.507a
Zn（120DAF）	4.76±0.576a	3.497±0.256a	5.993±0.508a	4.99±0.572a
Zn（130DAF）	5.45±0.52a	3.277±0.119a	4.623±0.144a	3.163±0.671a
Zn（140DAF）	3.337±0.275a	3.207±0.225a	2.763±0.545a	5.093±0.72a
Se（100DAF）	0.032±0.005a	0.008±0.001b	0.007±0.002b	0.029±0.003a
Se（110DAF）	0.007±0.003a	0.007±0.002a	0.01±0.003a	0.007±0.003a
Se（120DAF）	0.002±0.001a	0.008±0.01a	0.004±0.002a	0.031±0.003a
Se（130DAF）	0.009±0.003a	0.01±0.003a	0.009±0.003a	0.009±0.002a

Se（140DAF）	0.01±0.003a	0.011±0.003a	0.011±0.004a	0.01±0.002a

Parameter values shown in the table are expressed as mean ± standard error (n = 3). Different letters indicate statistical differences between treatments (Duncan test, $p < 0.05$).

Table S3. Fruit phenology and sugar and acid profiles of ‘liuyuezao’ pummelo after spraying with Ca or B and its mixture.

	**CK**	**Ca**	**B**	**Ca+B**
Single fruit weight（g）	2018±21.656a	2034.666± 17.097a	2009.333±6.658a	2046±47.843a
longitudinal diameter (cm)	20.246±0.456a	20.693±0.41a	20.873±0.12a	20.05±0.286a
diameter of the cross-section (cm)	18.4±0.264a	18.086±0.115a	18.943±0.431a	18.333±0.502a
fruit shape index	1.1±0.036a	1.144±0.021a	1.102±0.028a	1.094±0.045a
Su(mg/gFW)	31.724±0.093c	32.478±0.347c	32.922±0.418ab	33.435±0.462a
Fr (mg/gFW)	21.037±0.24d	21.716±0.135c	22.257±0.158b	22.652±0.205a
Glu (mg/gFW)	20.297±0.232b	20.468±0.148b	20.656±0.072b	21.076±0.255a
TS(mg/gFW)	73.426±0.398c	74.861±0.343b	75.622±0.478ab	75.885±0.498a
Cia (mg/gFW)	3.632±0.045a	3.52±0.078a	3.36±0.043b	3.221±0.068c

Maa (mg/gFW)	0.701±0.02a	0.574±0.01d	0.661±0.022b	0.618±0.011bc
Vc(mg/gFW)	0.663±0.007c	0.663±0.012c	0.737±0.012b	0.793±0.02a
Sha (ug/gFW)	5.348±0.132a	4.738±0.085b	4.585±0.025b	4.198±0.14c
Qua (ug/gFW)	47.777±0.962d	88.333±2.886a	73.888±1.924b	67.222±3.469c
α-Kea (ug/gFW)	4.69±0.52d	8.659±0.569a	6.956±0.536b	5.781±0.624bc
Ora (mg/gFW)	5.054±0.029a	4.859±0.066b	4.844±0.07b	4.709±0.06c
Gra	14.527±0.152c	15.407±0.239b	15.613±0.3b	16.113±0.208a

Parameter values shown in the table are expressed as mean ± standard error (n = 3). Different letters indicate statistical differences between treatments (Duncan test, $p < 0.05$).
